# Efficient induction and rapid identification of haploid grains in tetraploid wheat by editing genes *TtMTL* and pyramiding anthocyanin markers

**DOI:** 10.3389/fpls.2024.1346364

**Published:** 2024-03-19

**Authors:** Yanan Chang, Huali Tang, Surong Wang, Xi Li, Peipei Huang, Jiahui Zhang, Ke Wang, Yueming Yan, Xingguo Ye

**Affiliations:** ^1^ Institute of Crop Sciences, Chinese Academy of Agricultural Sciences, Beijing, China; ^2^ Beijing Key Laboratory of Plant Gene Resources and Biotechnology for Carbon Reduction and Environment Improvement, College of Life Science, Capital Normal University, Beijing, China

**Keywords:** durum wheat, *TtMTL* genes, CRISPR/Cas9, haploid induction, purple embryo

## Abstract

Doubled haploid (DH) technology provides an effective way to generate homozygous genetic and breeding materials over a short period of time. We produced three types of homozygous *TtMTL* gene-edited mutants (*mtl-a*, *mtl-b*, and *mtl-ab*) by CRISPR/Cas9 in durum wheat. PCR restriction enzymes and sequencing confirmed that the editing efficiency was up to 53.5%. The seed-setting rates of the three types of mutants ranged from 20% to 60%. Abnormal grain phenotypes of kernel, embryo, and both embryo and endosperm abortions were observed in the progenies of the mutants. The average frequency of embryo-less grains was 25.3%. Chromosome counting, guard cell length, and flow cytometry confirmed that the haploid induction rate was in the range of 3%–21% in the cross- and self-pollinated progenies of the *mtl* mutants (*mtl-a* and *mtl-ab*). Furthermore, we co-transformed two vectors, *pCRISPR/Cas9-MTL* and *pBD68-*(*ZmR + ZmC1*), into durum wheat, to pyramide *Ttmtl*-edited mutations and embryo-specifically expressed anthocyanin markers, and developed a homozygous durum haploid inducer with purple embryo (DHIPE). Using DHIPE as the male parent to be crossed with the wild-type Kronos, the grains with white embryos were identified as haploid, while the grains with purple embryos were diploid. These findings will promote the breeding of new tetraploid wheat varieties.

## Introduction

The haploid breeding technique has outstanding advantages over conventional breeding methods, which may shorten the breeding cycles by accelerating the stabilization of a homozygous genotype. Since the identification of haploid plants in jimsonweed (*Datura stramonium* L.) in 1921 (Blakeslee et al., 1922), a series of spontaneously generated haploids has been discovered (Weyen, 2021; Eliby et al., 2022). The means of naturally generating haploid plants include parthenogenesis, patrogenesis, and apogamy. However, these methods are not efficient in producing haploid plants on a large scale (Karimi-Ashtiyani, 2021; Eliby et al., 2022). Many methods for artificially inducing haploids have been developed, such as anther culture, ovule culture, microspore culture, and chromosome elimination using a wide cross and haploid inducer (HI). These strategies significantly increase the frequency of haploid production (Castillo et al., 2021; Karimi-Ashtiyani, 2021; Sari and Solmaz, 2021). In particular, the doubled haploid (DH) technology based on *in vivo* HI is widely used to accelerate breeding programs in maize (*Zea mays* L.) (Ishii et al., 2016). HI was only identified in maize germplasms but not in any other plant species. Therefore, it is necessary to create HI using novel technologies for crops that require HI.

Genome-editing technology is becoming more prevalent in plants and animals. Specific nucleases are used to edit and modify targeted DNA sites in the genome. Clustered regularly interspaced short palindromic repeats (CRISPR) are a type of bacterial defense system that degrade alien DNA. It interacts with various CRISPR-associated proteins (Cas9) (Bao et al., 2019). In the CRISPR/Cas9 system, Cas9 and single guide RNA (sgRNA) form a chimeric proteome, and Cas9 recognizes the simple PAM sequence 5’-NGG-3’ of the genomic DNA directed by sgRNA and cuts the double-stranded DNA. Using CRISPR/Cas9 editing technology, mutations in plant target genes can be generated when broken DNA strands are repaired via non-homologous terminal junctions or homologous recombination (Puchta et al., 1996). Compared with other genome-editing technologies, CRISPR/Cas9 is preferred because of its simplicity in vector construction, high editing efficiency, and low off-target efficiency (Wu and Yin, 2019). To date, CRISPR-Cas9 has been applied in many plants like *Arabidopsis thaliana*, tobacco (*Nicotiana tabacum* L.), wheat (*Triticum aestivum* L.), maize, rice (*Oryza sativa* L.), barley (*Hordeum vulgare* L.), soybean (*Glycine max* L.), and tomato (*Solanum lycopercicum* L.) (Li et al., 2013; Shimatani et al., 2017; Zong et al., 2017; Gan and Ling, 2022).

Haploid induction in maize is triggered mainly by *MATRILINEAL* (*MTL*) or *PLA/NLD*, a pollen-specific phospholipase. The frameshift mutation *MTL/PLA/NLD* can produce a haploid induction rate (HIR) of 6.7% in maize (Kelliher et al., 2017; Liu et al., 2017). By referring to the sequences of maize *MTL/PLA/NLD*, many mutants for haploid induction have been developed in other plants using genome editing. The loss of function mutations in wheat *MTL*/*PLA1* caused by genome editing can directly induce maternal haploids with an HIR of 5.88%–15.66% in different types of mutations, including *TaPLA-A/TaPLA-D*, *mtl-AD*, *mtl-BD*, and *mtl-ABD* in self- or cross-pollinated combinations (Liu et al., 2020a; Liu et al., 2020b; Tang et al., 2022). The HIRs induced by *MTL/NLD* edited mutants were reported to be 2%–6% in rice (Yao et al., 2018) and 2.8% in fox millet (*Setaria italica* L.) (Cheng et al., 2021).

In maize HI, genes encoding phospholipase D (*ZmPLD*) and a domain of unknown function 679 membrane protein (*ZmDMP*) were also associated with the induction of maternal haploids (Zhong et al., 2019; Li et al., 2021). Silent mutations of *ZmPLD3* via genome editing mediated by CRISPR/Cas9 resulted in HIR from 1.19% to 4.13%, which exhibited a synergistic effect with *Zmmtl/Zmpla1/Zmnld* rather than functional redundancy (Li et al., 2021). Loss of *AtDMP* function in *Arabidopsis* could trigger maternal haploid induction (Zhong et al., 2020). The average HIR triggered by *SlDMP* mutations from genome editing in tomato was 1.90% in crosses produced by 36 different female genotypes (Zhong et al., 2022). In potatoes (*S. tuberosum* L.), the *Stdm*p-triggered HI system developed by CRISPR/Cas9 was used to obtain homozygous diploid lines (Zhang et al., 2022). In watermelon (*Citrullus lanatus* L.), haploids can be achieved with efficiencies ranging from 0.55% to 1.08% by employing *ClDMP-*edited mutants as male parents to cross with other female parents (Tian et al., 2023). In cotton (*Gossipium hirsutum* L.), the *Ghdmp* inducer lines created by CRISPR can induce F_1_ hybrid females to generate haploids at a rate of 1.06% (Long et al., 2023).

Although haploid seeds can be induced in many plants using the HI developed via the genome editing strategy, it is difficult to directly discriminate haploid and diploid grains in crossing progenies. Generally, plant ploidy is identified through chromosome number, guard cell length, gene copy number, and total DNA amount at the morphological, cytological, and genomic levels. However, these methods are not convenient and efficient for breeding programs. In a recent study, maize haploid grains induced by *ZmMTL*-edited mutants were visually identified using green fluorescent protein (eGFP) and red fluorescent protein (DsRED) driven by maize embryo-specific and barley endosperm-specific promoters, respectively (Dong et al., 2018). More conveniently, the haploid maize grains were directly distinguished by embryo color. Maize HI was labeled with embryo-specifically expressed *ZmR2* and *ZmC1* driven by the embryo-aleurone-specific bidirectional promoter (Chen et al., 2022). Using a similar strategy, wheat haploid grains without anthocyanin markers induced by embryos specifically labeled with *TaMTL*-edited mutants with *ZmC1* and *ZmR* were also conveniently visualized accurately (Qi et al., 2023; Tang et al., 2023).

Durum wheat (*T. turgidum* subsp. *durum*, AABB, 2n = 4x = 28) is cultivated second only to common wheat. This crop has high gluten and protein contents but it is low in yield, and susceptible to *Fusarium* head blight (caused by *F. gramminum*) and root rot (caused by *Bipolaris sorokiniana*). Compared with common wheat, durum wheat has only genomes A and B. It is easier to cross with wild relatives to develop germplasms containing alien chromosomes in their genetic backgrounds (Mulugeta et al., 2023). To date, haploid plants in durum wheat have mainly been induced by microspore culture and intergeneric crossing between tetraploid wheat and maize (Cistué et al., 2009). However, efficient induction and rapid identification techniques for haploids in durum wheat have not yet been reported. The aim of this study was to create an HI line in durum wheat by editing the *TtMTL* gene using CRISPR/Cas9, and then pyramiding the *TtMTL* mutations and anthocyanin genes of *ZmC1* and *ZmR* using the co-transformation strategy. The results obtained in the current study will promote haploid breeding of tetraploid wheat.

## Materials and methods

### Plant materials and growth condition

The tetraploid durum wheat variety, Kronos, was used for genetic transformation and genome editing. Seeds were planted in pots (20 cm in diameter and 30 cm in height) and maintained in a growth chamber at 25°C/18°C (day/night) with a photoperiod of 16 h/8 h (light/dark), 300 μmol m^−2^ s^−1^ light intensity, and a relative humidity of 45% to collect immature grains for use in genetic transformation. During the growth period of the mother plants, aphids (*Aphidoidea*) were prevented using sticky colored cards (Zhengzhou Oukeqi Instrument Co. Ltd., Zhengzhou, China), and powdery mildew (PM, caused by *Blumeria graminis* f. sp. *tritici*) were controlled by the application of Triadimefon (Jinan Luba Pesticides Co., Jinan, China).

### Vector construction for editing *TtMTL* and expressing *ZmC1* and *ZmR*

The expression vector, *pWMB-110-SpCas9*, was used for genome editing. sgRNAs of *TtMTL* were incorporated into the vector, following a previously described method (Liu et al., 2020b). Two targets for the two durum wheat genes *TtMTL-4A* (TRITD4Av1G005320) and *TtMTL-4B* (TRITD4Bv1G168750) were selected according to their coding sequences (CDS) in WheatOmics (http://202.194.139.32). Based on the requirements for sgRNA upstream of a PAM motif (5’-NGG-3’) and mutation detection by restriction enzymes, two 20-bp sgRNA sequences (5’-AGCTGCAGGAGCTGGACGGC-3’ and 5’-CGGTGACCGCATCGCTGAGG-3’) were designed to construct the new vector *pCRISPR/Cas9-MTL*. Another expression vector, *pBD68-(ZmR + ZmC1)*, was used to generate transgenic durum wheat plants in which *ZmR* and *ZmC1* were specifically expressed in embryo tissues (Tang et al., 2023). The two constructed vectors were further verified by sequencing and then transformed into the *Agrobacterium tumefaciens* strain GV3101.

### *Agrobacterium*-mediated transformation of durum wheat

Immature durum wheat grains with immature embryos 2 mm–3 mm in diameter were collected at 14 d–16 d post-anthesis (DPA), sterilized with 75% ethanol for 1 min and 15% sodium hypochlorite for 5 min, and washed five times with sterile water under aseptic conditions. Immature embryos were isolated and infected with *Agrobacterium* cells harboring the constructed vectors to generate transgenic plants. The genetic transformation of durum wheat was performed according to a previously published protocol (Wang et al., 2017). Briefly, isolated immature embryos were collected in a 2.5 mL tube containing 2 mL of WLS liquid medium [1/10 Linsmaier and Skoog (LS) salts, 1/10 Murashige and Skoog (MS) vitamins, glucose 10 g L^−1^, 2-(N-morpholino) ethanesulfonic acid (MES) 0.5 g L^−1^, and acetosyringone (AS) 100 μM, pH 5.8], centrifuged at 4°C for 10 min at 7,500×*g*, and incubated in the same volume of *Agrobacterium* solution in WLS for 10 min at room temperature. *Agrobacterium*-infected embryos were placed onto the co-cultivation medium (WLS plus AgNO_3_ 0.85 mg L^−1^, CuSO_4_·5H_2_O 1.25 mg L^−1^, and agarose 8 g L^−1^) at 23°C in darkness. Embryonic axes of the infected tissues were removed and the remaining scutella were cultured on rest medium (containing LS salts, MS vitamins, 2,4-D 0.5 mg L^−1^, picloram 2.2 mg L^−1^, AgNO_3_ 0.85 mg L^−1^, ascorbic acid 100 mg L^−1^, carbenicillin 250 mg L^−1^, cefotaxime 100 mg L^−1^, MES 1.95 g L^−1^, and agarose 5 g L^−1^) at 25°C for 5 d in the dark. Next, the tissues were transferred onto selection medium [rest medium plus phosphinothricin (PPT), Sigma, no. 45520, with 5 mg L^−1^ and 10 mg L^−1^] for 2 weeks and 3 weeks at 25°C, respectively. Finally, embryonic calli were transferred to differentiation medium containing 5 mg L^−1^ PPT at 25°C with a daily interval of 100 μmol m^–2^ s^–1^ light for 14 h and dark for 10 h. Regenerated green shoots were transferred to a rooting medium containing 5 mg L^-1^ PPT. Transgenic plants (5 cm–8 cm in height) with well-developed roots were transplanted into pots and cultivated in a growth chamber under the same conditions as mentioned above.

### Molecular detection of transgenic durum wheat plants

Genomic DNA was extracted from the leaf tissues of T_0_ transgenic plants using a Nuclear Plant Genomic DNA Kit (CW Bio Inc., Taizhou, China). Specific primers for *bialaphos resistance* (*bar*), editing targets, *ZmC1*, *TtMTL*-*4A*, and *TtMTL*-*4B* genes were designed to detect alien integrations or mutations by PCR, digestion, and sequencing (**Table 1**). Genes *TtMTL*-*4A* and *TtMTL*-*4B* in positive transgenic plants were first amplified by PCR in a T100TM Thermal Cycler (Bio-Rad, Hercules, CA, USA) using a program that included an initial denaturation at 95°C for 5 min, 35 cycles of 30 s at 95°C, 30 s at 60°C, and 60 s at 72°C, and a final extension at 72°C for 8 min. The mutation types of *TtMTL*-*4A* and *TtMTL*-*4B* were detected by PCR restriction enzyme (PCR-RE) assay in a 20 μL reaction system for 2 h at 37°C. The digested PCR products were separated on a 1.5% agarose gel and visualized using Genecolor DNA Staining II TM (Gene-Bio Ltd., Beijing, China) to verify the editing type according to the size and number of the digested bands.

**Table 1:** PCR primers used in this study.

Primers	Primer sequence (5’- 3’)
Bar-F	ACCATCGTCAACCACTACATCG
Bar-R	GCTGCCAGAAACCACGTCATG
TtMTL-4A-F	GCCGAGACTTCACTTACGCT
TtMTL-4A-R	CCATCGTCTGCGTTTGTCAC
TtMTL-4B-F	TGAACTCAACATGGGGCGTC
TtMTL-4B-R	TCAGTTGGTCAGTGTGCTCG
ZmR-F	ATGGCGCTTTCAGCTTCCC
ZmR-R	TCACCGCTTCCCTATAGCTTTGC

### Cytological and histological detection of the *TtMTL*-edited mutations

The chromosomes in the root tips of durum wheat plants were examined using a previously published method (Han et al., 2004; Yuan et al., 2015). First, fresh roots 1 cm–2 cm in length were collected from germinated *TtMTL-*edited T_1_ seeds, treated with nitrous oxide for 2 h, fixed in 90% acetic acid (v/v) for 5 min, and washed three times with sterile water. Next, the root tips were digested in enzymatic hydrolysate for 1 h. Chromosomes in mitotic metaphase were observed under a light microscope (BX51; OLYMPUS, Tokyo, Japan). Leaf pieces of 2 cm in length were collected from the *TtMTL*-edited plants at the jointing stage and placed on glass slides with the abaxial side facing up. Mesophyll tissues were removed using a sharp knife. Guard cells were observed under a light microscope. Anthers were sampled and stained with 1% I_2_-KI solution for 10 min. Pollen fertility was observed under a light microscope. The ploidy levels of the selected plants, identified by chromosomes or guard cells, were confirmed by flow cytometry at Ploidy Expert Biotechnology Co., Ltd. (Beijing, China).

## Results

### Generation of the *TtMTL*-edited plants in durum wheat

**Figure 1:**
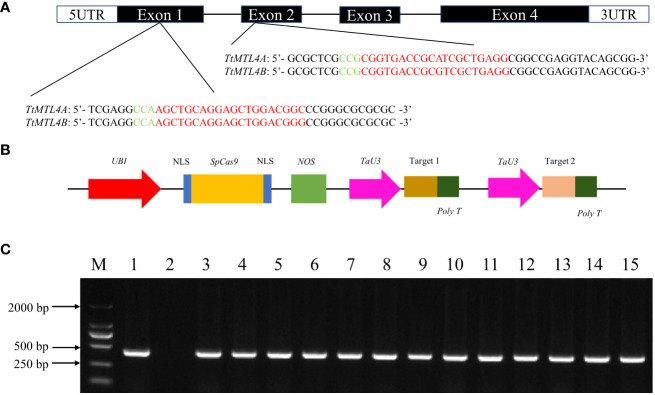
Target site design for editing *TtMTL* genes and detection of edited/transgenic durum wheat plants. **(A)** Coding frame and target sequences of *TtMTL* genes. Green and red letters indicate the PAM and sgRNA sequences, respectively. **(B)** CRISPR/SpCas9 vector structure for editing *TtMTL*. **(C)** Detection of *bar* genes in T_0_ edited/transgenic plants. 1: Positive control. 2: wild-type. 3–15: T_0_ edited/transgenic plants. M: DNA ladder DL 2000.

The *TtMTL* gene in durum wheat contains four exons and three introns according to the cDNA and gDNA sequences on chromosomes 4A (chr4A_Durum_Wheat) and 4B (chr4B_Durum_Wheat). To edit *TtMTL-4A* and *TtMTL-4B* in durum wheat using the CRISPR/SpCas9 system, two sgRNA sequences targeting the first and second exons, respectively, were designed based on the conserved sequences of the target genes (**Figures 1A, B**). In total, 80 immature embryos collected from the Kronos genotype were transformed with *Agrobacterium* containing the CRISPR/SpCas9 vector to edit the two *TtMTL* genes. After differentiation and regeneration cultures on selection medium for approximately three months, 120 transgenic or edited plants were obtained and tested for the presence of the *bar* gene by PCR (**Figure 1C**).

**Figure 2:**
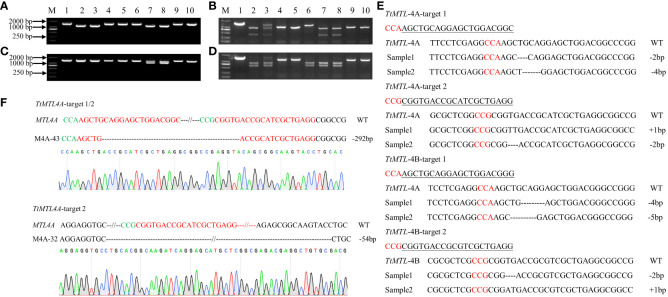
Edited-type detection of *TtMTL* genes in T_0_ edited/transgenic durum wheat plants by PCR, enzyme digestion and sequencing. **(A)** PCR products of *TtMTL-4A* in the edited/transgenic plants. 1–9: PCR products of *TtMTL-4A*, 10: PCR products of *TtMTL-4A* in wild-type (WT). **(B)** Detection of edited types within the *TtMTL-4A* gene in T_0_ transgenic plants using *Pst*I enzyme digestion. 1: Undigested PCR products from the WT. 2: Digested PCR products in the wild-type. 3–10: Digested PCR products of *TtMTL-4A* in transgenic plants. **(C)** PCR products of *TtMTL-4B* in edited/transgenic plants. 1–9: PCR products of *TtMTL-4B*. 10: PCR products of *TtMTL-4B* in the WT. **(D)** Detection of edited types within the *TtMTL-4B* gene in T_0_ transgenic plants by *Pst*I enzyme digestion. 1: Undigested PCR products in wild-type. 2: Digested PCR products in the WT. 3–10: Digested PCR products of *TtMTL-4B* in transgenic plants. **(E)** InDel mutation detection in the two *TtMTL* homoeologous genes in the T_0_ generation by DNA sequencing. Red letters indicate the PAM sequences. Nucleotide deletions are displayed as dashed lines. **(F)** Sequencing analysis of *TtMTL-4A* between the two target sites. Green and red letters indicate the PAM and sgRNA sequences, respectively. The dashed lines represent nucleotide deletions. The size of the deletion fragment is shown on the right-hand side.

Based on the results of PCR-RE, the editing efficiencies of the *TtMTL* gene at targets 1 and 2 were 43.2% and 34.3%, respectively. The total editing efficiency was 53.5% in transgenic plants in which a mutation occurred at one or two sites (**Figures 2A–D**; **Table 2**). The editing efficiencies of *TtMTL-4A* and *TtMTL*-*4B* targeting the first exon were 33.3% and 20.8%, respectively, and those targeting the second exon were 31.6% and 15.8%, respectively (**Table 2**). Among the confirmed edited plants, 18 plants were edited in *TtMTL-4A* and *TtMTL*-*4B* simultaneously, with an editing efficiency of 15.0%.

**Table 2:** Analysis of different editing types of *TtMTL* at the two target sites in the T_0_ transgenic plants.

Target loci	PAM-guide sequence (5’-3’)	Transgenic plants	Mutant plants	Mutation rate (%)	Genotype	LFD	LFDR (%)
*TaMTL*-4A-target 1	CCAAGCTGCAGGAGCTGGACGGC	120	40	33.3	13Bi + 27He + 80WT	11	9.2
*TaMTL*-4A-target 2	CCGCGGTGACCGCATCGCTGAGG		38	31.6	10Bi + 28He + 82WT		
*TaMTL*-4B-target 1	CCAAGCTGCAGGAGCTGGACGGC	120	25	20.8	8Bi + 17He + 95WT	5	4.2
*TaMTL*-4B-target 2	CCGCGGTGACCGCATCGCTGAGG		19	15.8	6Bi + 13He + 101WT		

Bi, bi-allele; He, heterozygote; WT, wild-type; LFD, large fragment deletion; LFDR, large fragment deletion rate.

The DNA sequencing results from the edited plants showed that the mutation types included nucleotide insertions and deletions (**Figure 2E**). The mutation types within *TtMTL-4A* were dominated by deletions of 2 bp or 4 bp and insertion mutations of 1 bp at the two targets. The mutation types within *TtMTL-4B* at the two targets mainly included 2-bp, 4-bp, and 5-bp deletions, and 1-bp insertions. There was a large fragment deletion between the two targets in one mutant, in which a 292 bp fragment deletion was detected in both the edited *TtMTL-4A* and *TtMTL-4B*, and another 54-bp deletion was only found in the edited *TtMTL-4A* (**Figure 2F**; **Table 2**). The two targets were simultaneously edited in either *TtMTL-4A* or *TtMTL*-*4B* with efficiencies of 9.2% and 4.2%, respectively.

### Phenotypic identification of the *TtMTL*-edited plants in durum wheat

**Figure 3:**
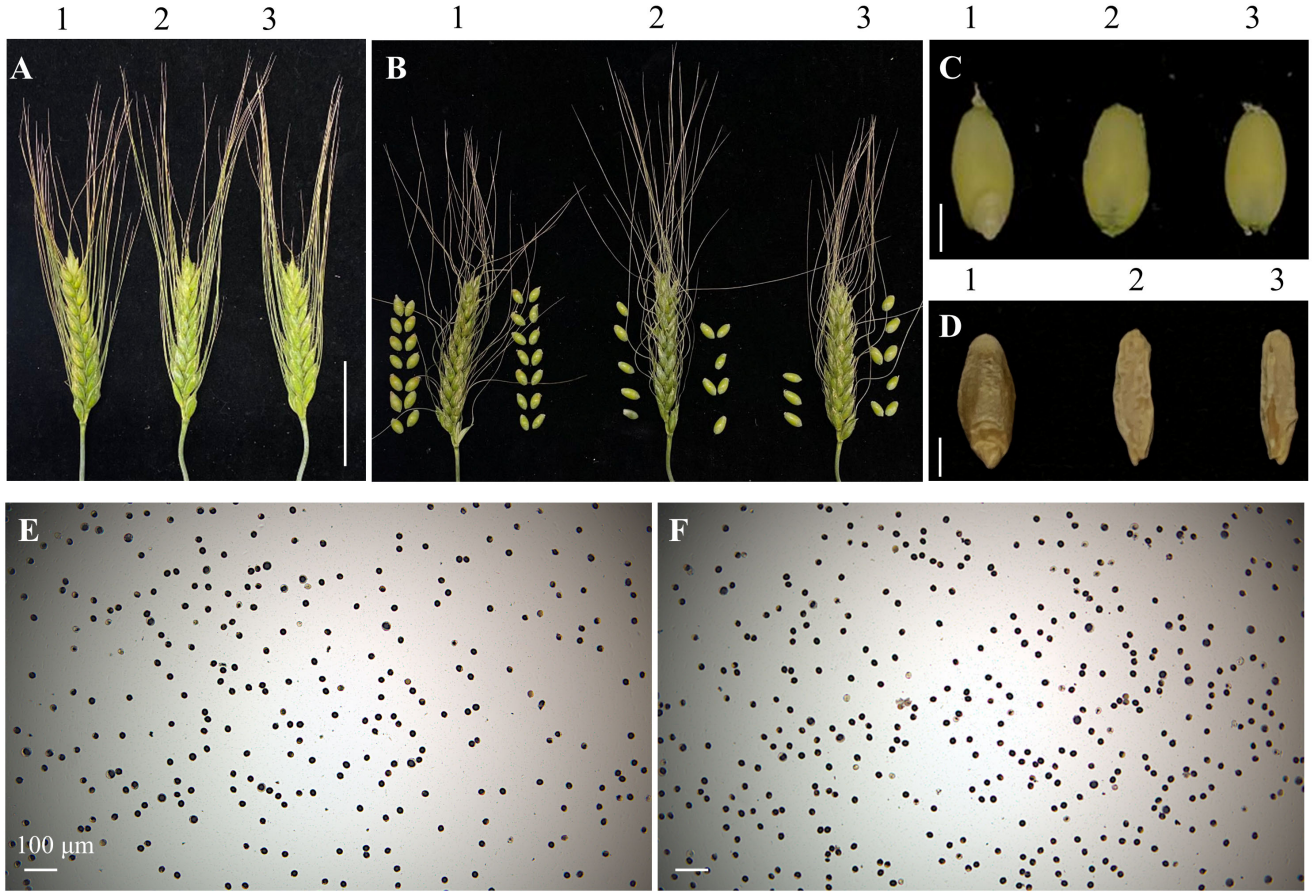
Spike and grain phenotypes and pollen viability of the *TtMTL*-edited plants. **(A)** Spikes. 1: The Wild-type (WT). 2–3: *TtMTL*-AB edited plants. **(B)** Spikes accompanying with threshed grains. 1: The WT. 2–3: *TtMTL*-AB edited plants. Scale bar = 1 cm. **(C)** No embryo grains. 1: The WT. 2–3: No embryo grains of the *TtMTL*-*AB* edited plants. **(D)** Empty grains. 1: The WT. 2-3: No embryo and endosperm grains of the *TtMTL*-*AB* edited plants. Scale bar = 3 mm **(E)** Pollen viability assay with KI staining for the WT. **(F)** Pollen viability assay with I_2_-KI staining for the *TtMTL*-edited plants. Scale bar = 100 μm.

All T_0_ transgenic and edited plants, as well as their wild-type (WT) plants, were grown in a greenhouse. During the entire growth period, no obvious difference in botanic traits was found between transgenic/edited and WT plants, except for the seed setting rate (SSR). The SSR of the *TtMTL*-edited plants ranged from 20% to 60%, which was significantly lower than that of WT (**Figures 3A, B**). Plants in which *TtMTL*-4A and *TtMTL*-4B were edited simultaneously showed an average SSR of 20.5%. Plants in which *TtMTL*-4A or *TtMTL*-4B were edited only showed SSR of 32.3% and 58.2%, respectively. The WT had an SSR of 94.6% (**Table 3**). In particular, embryoless grains (ELG) were observed in the progenies of the three types of *TtMTL*-edited plants (*mtl-a*, *mtl-b*, and *mtl-ab*) with an average frequency of 25.3% (**Figure 3C**; **Table 3**). In addition, seeds with seed coats without embryos or endosperms were also found in the progenies of the edited plants (**Figure 3D**). Further investigation revealed that the pollen viability of the edited plants was similar to that of the WT plants after staining with I_2_-KI (**Figures 3E, F**).

**Table 3:** Analysis of grain phenotypes in the self-and cross-pollinated combinations of the *Ttmtl* mutants.

Female parent	Male parent	Seed setting rate (%)	Haploid induction rate (%)	Number of normal grains	Number of embryo-less grains	Frequency for embryo-less grains (%)
*mtl-a*	Ⓧ	32.3	3.2	200	32	16.0
*mtl-b*	Ⓧ	58.2	0	180	16	8.0
*mtl-ab*	Ⓧ	20.5	20.6	220	48	21.8
Kronos	*mtl-a*	38.1	0	80	15	18.8
Kronos	*mtl-b*	56.6	0	75	9	12.0
Kronos	*mtl-ab*	24.4	18.3	102	20	19.6

Ⓧ, self-cross.

### Cytological and histological identification of haploids in the progenies of *TtMTL*-edited durum wheat mutants

**Figure 4:**
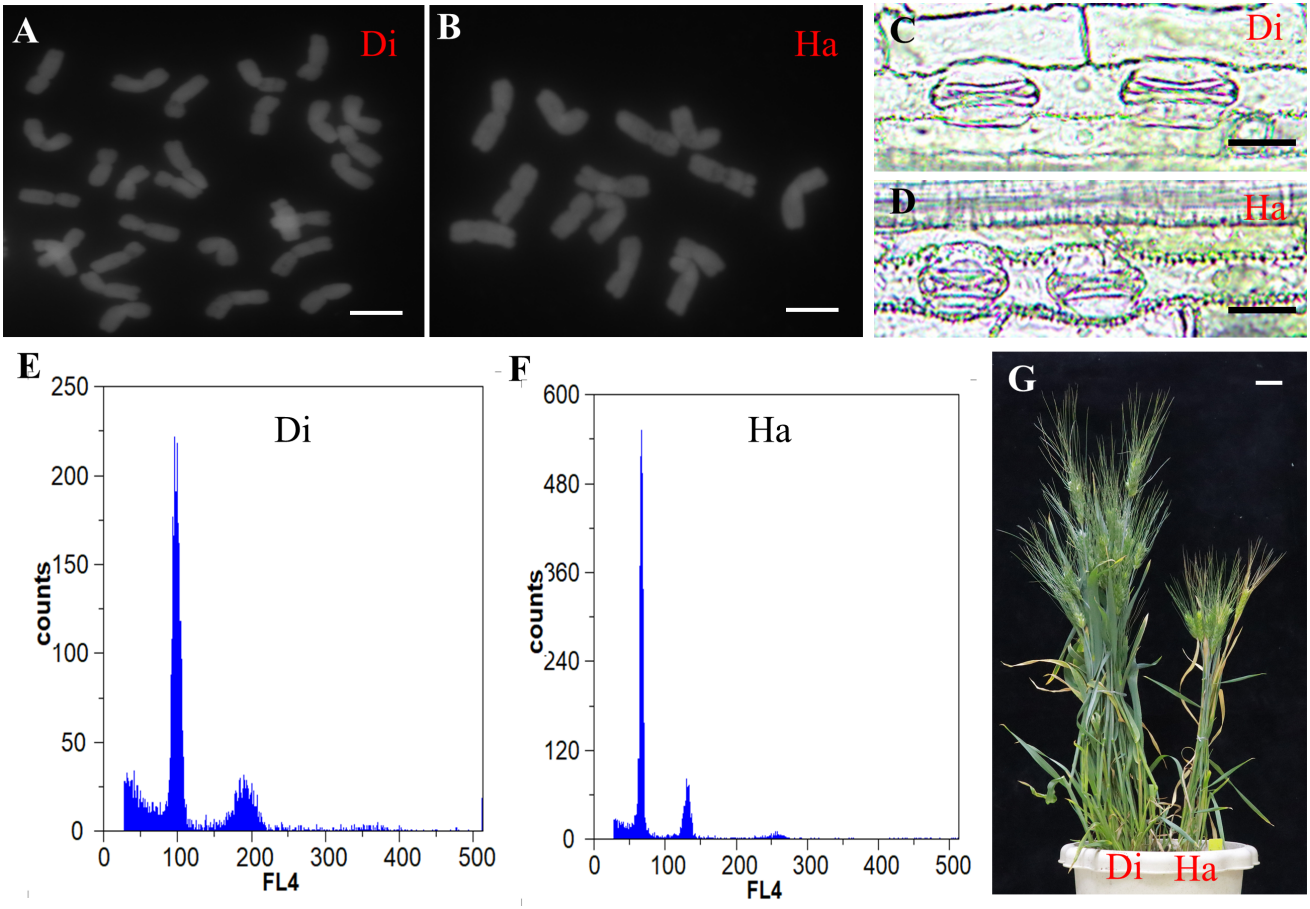
Cytological and histological identification of haploid plants in the *TtMTL*-edited progenies. **(A)** Chromosomes diploid. **(B)** Chromosomes of haploid. Scale bar = 10 μm. **(C)** Guard cells of diploid plants. **(D)** Guard cell length of haploid plants. Scale bar = 30 μm. **(E)** Flow cytometry analysis of diploid plant. **(F)** Flow cytometry analysis of haploid plant. **(G)** Adult plants of the *TtMTL*-edited plants, diploid plant in the left side and haploid plant in the right side. Scale bar = 5 cm.

In total, 120 seeds harvested from *TtMTL-*edited plants were examined for chromosome number. Consequently, 20 haploid grains are identified. According to the cytological identification results, the haploid grains had only 14 chromosomes, while the remaining diploid grains had 28 chromosomes (**Figures 4A, B**). At the jointing stage, the average guard cell length was 39.5 μm in haploid plants and 58.3 μm in diploid plants (**Figures 4C, D**). At the grain-filling stage, haploid plants displayed shorter plant height, fewer spikes per plant, narrower and shorter leaves, and smaller spikes than diploid plants (**Figure 4E**). In the flow cytometry analysis, diploid plants showed a 100 FL4 peak and haploid plants showed a 50 FL4 peak (**Figures 4F, G**). Combining cytological and histological identification, the HIR was 3%–21% among the three types of mutants (**Table 3**). No haploid grains were found in *mtl-b*-mutant material. The HIR of the *mtl-a* mutant was significantly lower than that of the *mtl-ab* mutant (**Table 3**).

### Development of durum HI with purple embryo contributed by the tissue specific expression of *ZmR* and *ZmC1*

**Figure 5:**
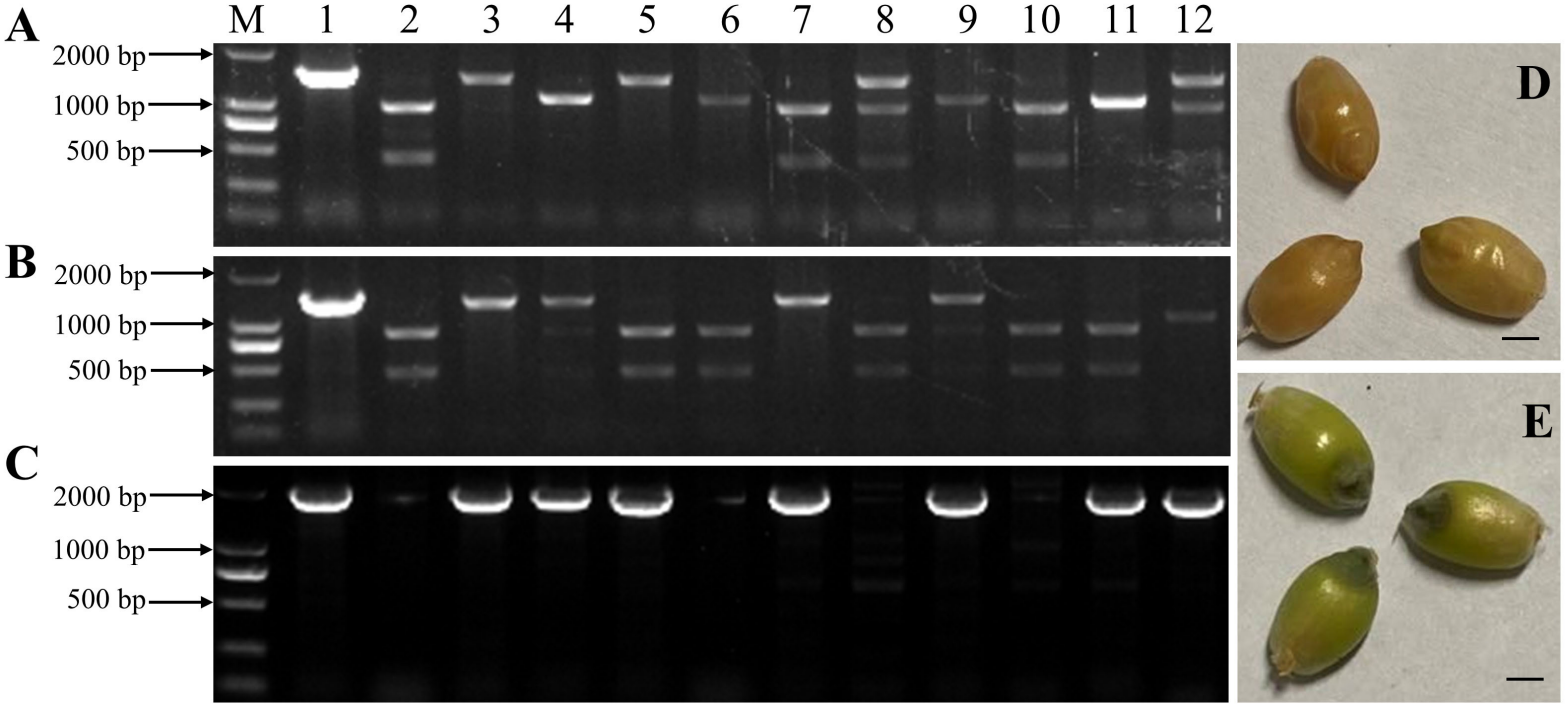
Development and identification of the *TtMTL*-edited haploid inducer labeled with embryo-specifically expressed genes *ZmR* and *ZmC1* in durum wheat. **(A)** Detection of the edited *TtMTL-4A* in edited/transgenic plants from the co-transformation of *pCRISPR/Cas9-MTL* and *pBD68-(ZmR + ZmC1)*. 1: Undigested PCR products of *TtMTL-4A* in the wild-type (WT). 2: Digested PCR products of *TtMTL-4B* in the WT. 3-12: *Pst*I digested PCR products of *TtMTL-4A* in the edited/transgenic plants. **(B)** Detection of edited *TtMTL-4B* in the edited/transgenic plants. 1: Undigested PCR products of *TtMTL-4B* in the WT. 2: Digested PCR products of *TtMTL-4B* in the WT. 3–12: *Pst*I digested PCR products of *TtMTL-4B* in the edited/transgenic plants. **(C)** Detection of genes *ZmR* and *ZmC1* in the edited/transgenic plants. 1: Positive control. 2: The WT. 3–12: T_0_ transgenic plants. M: DNA ladder DL 2000. **(D)** The WT grains with white embryos at the grain-filling stage. **(E)** The edited/transgenic grains with purple embryos at the grain-filling stage.

**Figure 6:**
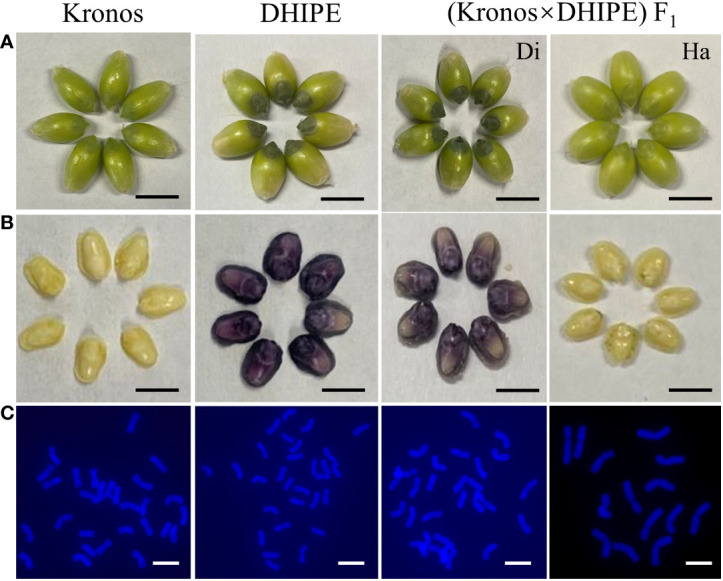
Grains and embryos with white and purple colors from different durum wheat materials and their cytological confirmation for ploidy. **(A)** Grain phenotype in the wild type (WT) durum wheat variety Kronos, durum haploid inducer with purple embryo (DHIPE), and the hybrids from the cross of Kronos × DHIPE. Scale bar = 5 mm. **(B)** Grain phenotype in Kronos, DHIPE, and the hybrids from the cross of Kronos × DHIPE. Scale bar = 2 mm. **(C)** Chromosome counting in Kronos, DHIPE, and the hybrids from the cross of Kronos × DHIPE. Di, diploid grains from Kronos × DHIPE. Ha, haploid grains from Kronos × DHIPE. Scale bar = 5 μm.

To develop a rapid method for the visual identification of haploid durum wheat seeds induced by HI mutants at the grain stage, we co-transformed Kronos with vectors *pCRISPR/SpCas9-MTL* and *pBD68-(ZmR + ZmC1)* for pyramiding *Ttmtl*-edited mutations and embryo-specifically expressed anthocyanin markers. In total, 80 immature durum wheat embryos were infected with *Agrobacterium* to produce 110 putative transgenic plants. All were positive for the *bar* gene by PCR. Among them, eight transgenic/edited plants were simultaneously edited at *TtMTL-4A* and *TtMTL-4B* (*mtl*-*ab*), which carry the two anthocyanin genes (**Figures 5A–C**). During the grain-filling stage, we found that the transgenic plants containing the *pBD68-(ZmR + ZmC1)* expression cassette (TRC) exhibited a dark purple color only in the embryos, whereas the WT exhibited a normal color (**Figures 5D, E**, **6A**). After self-crossing the transgenic and edited plants three times, a homozygous durum haploid inducer material with purple embryos (DHIPE) was developed.

### Application of DHIPE in the induction and reorganization of haploid durum grains

To test the effectiveness of DHIPE in the induction and recognition of haploid durum grains based on embryo color, DHIPE was crossed with wild-type Kronos as male parent (**Figures 6A, B**). A total of 146 hybrid grains were obtained from the cross, of which 36 were embryo-less (25.3%). In the remaining 110 normal grains, 14 grains showed white embryos, and 96 grains showed purple embryos. Cytological observations indicated that all grains with purple embryos were diploid with 28 chromosomes, but those with white embryos were haploid with 14 chromosomes (**Figure 6C**), and the HIR in the hybrids was 12.7%. These results suggested that the accuracy of ploidy identification according to embryo color using DHIPE as a male parent was almost 100%.

## Discussion

The technology of haploid chromosome doubling provides an effective way to accelerate the production of homozygous inbred lines in crop breeding and thus promotes the development of new varieties or germplasms. The production of plant haploids mainly relies on *in vitro* culture and parthenogenesis. However, there is a strong genotype preference for generating haploids by anther and microspore cultures (Forster et al., 2007). Even though the generation of haploids by parthenogenesis, including pollen radiation, ovarian treatment with chemicals, and distant hybridization, is less efficient, it is tissue culture-independent. Fortunately, haploid induction materials have been found in maize that can easily induce female parents to produce haploid grains as pollen donors. This induction method has been widely used in maize breeding.

With the identification and cloning of genes related to haploid induction in maize HI, breeding haploid induction materials using genome editing has become an important technique for plant improvement (Jacquier et al., 2020). This strategy has been extended to other crops because it is easy to implement (Meng et al., 2021). For example, when the homologous gene *TaPLA/TaMTL* in wheat of *ZmMTL/ZmPLA1/ZmNLD* in maize was knocked out using CRISPR/Cas9, haploid grains could be induced in self-pollinated progenies with a frequency of 5%–32% in different edited combinations (Liu et al., 2020a; Liu et al., 2020b). The HIR of *Tamtl-abd* and *Tamtl-ad* were 11.8%–31.6% and 10%, respectively. Because the centromere histone-encoded gene *TaCENH3a* was silenced by genome editing, haploid grains were obtained with an efficiency of 7% (Lv et al., 2020). We obtained three types of edited mutants by knocking out two *TtMTL* genes in durum wheat using CRISPR/Cas9. *mtl-ab* mutations produced a 20% HIR, which was much higher than that induced by single mutations in *mtl-a* (3%) or *mtl-b* (0). With regard to the homologous gene *TtMTL-A* in tetraploid wheat, *TtMTL-B* might be a potentially redundancy and functionally complementary *mtl-a* single mutation, resulting in a lower HIR than the double mutations of *mtl-ab*.

Two hypotheses have been proposed regarding the mechanism of haploid induction in maize. One is single fertilization, and the other is double fertilization or chromosome elimination (Tian et al., 2018; Kelliher et al., 2019). In the second hypothesis, it was thought that the two sperm cells fuse with the egg and the central cell, but the male chromosomes were selectively eliminated during early embryo development. We found that pollen viability between *TtMTL-*edited mutants and their WT was not different, which supports the second hypothesis.

In plants, reproductive abnormalities and segregation distortions of some traits are often caused by maternal haploid induction. In particular, embryo abortion, resulting in the formation of defective kernels, is present in the self-pollinated grains of HI and the cross-pollinated grains using HI as the male parent (Nair et al., 2017; Chaikam et al., 2018). In the self-pollinated generation of *mtl-ABD* mutants in hexaploid wheat, five types of grains, such as normal diploid grains, haploid grains with endosperms, haploid grains without endosperms, diploid grains without endosperms, and defective grains without endosperms and embryos, led to a much lower SSR of the mutant (Liu et al., 2020b; Tang et al., 2022). Embryo abortion and both embryo and endosperm abortion were found in the self- or cross-pollinated progenies of the *mtl-ab* mutants in durum wheat, and the SSR of the mutant was also very low. Our findings for tetraploid wheat are consistent with the results for hexaploid wheat (Tang et al., 2022).

A haploid inducer with a high HIR also triggers kernel abortion at a high frequency. However, there is no close correlation between endosperm abortion and HIR in HI (Chaikam et al., 2018). The developmental stages of embryo and endosperm abortion in HI have not yet been clearly clarified. We inferred that embryo abortion or endosperm abortion might occur during either fertilization or post-fertilization due to the failure of double fertilization, resulting in the suspension of zygote or endosperm development.

Recently, the identification of haploids has been simplified and visualized using eGPF proteins and DsRed signals specifically expressed in the embryo and endosperm (Dong et al., 2018). Subsequently, a visually fast technique was reported to recognize haploid grains in common wheat by labeling embryos of the *mtl-ABD* mutant with embryo-specifically expressed anthocyanin, in which the diploid grains had purple embryos and the haploid grains had white ones (Qi et al., 2023; Tang et al., 2023). We generated transgenic/edited plants of tetraploid wheat carrying two anthocyanin genes and an *mtl-ab* mutation by co-transformation. We further developed a new DHIPE material, in which the edited *TtMTL* genes and the two anthocyanin genes were pyramided. Using DHIPE as the male parent, the haploid and diploid grains in the hybrids can be visually distinguished easily by embryo color, which will definitely boost the haploid breeding of tetraploid wheat.

Generally, haploid plants induced by edited mutants cannot be naturally doubled, and chromosome doubling by artificial methods is usually required. Haploid seedlings developed from haploid immature embryos or seeds need to be treated with antimitotic activity chemicals, such as colchicine, herbicide, and nitrous oxide for chromosome doubling (Meng et al., 2021). However, the chromosome doubling method at the seedling stage is time-delayed, inconvenient, and inefficient. Additionally, the application of colchicine in chromosome doubling has some problems, including toxicity, environmental pollution, endangering human health, and high cost. In contrast, propyzamide might be an alternative to colchicine (Yin et al., 2018). In our future work, we plan to treat the haploid grains of durum wheat during the germination step with promising concentrations and treatment times of colchicine and propyzamide.

## Conclusions

We established an efficient haploid induction system by editing the *TtMTL* gene using CRISPR-Cas9 in durum wheat. The haploid induction rate of the edited mutations *mtl-a* and *mtl-ab* ranged from 3% to 21% in the self- and cross-pollinated progenies, whereas the *mtl-b* mutation was not able to trigger haploid generation. The *Ttmtl*-edited mutations and embryo-specifically expressed anthocyanin genes were pyramided together in a novel *mtl-ab* mutant DHIPE with purple embryos. By applying DHIPE as a male parent, haploid grains can be easily recognized based on embryo colors in the hybrids.

## Data availability statement

The original contributions presented in the study are included in the article/supplementary material, further inquiries can be directed to the corresponding author/s.

## Author contributions

XY: Funding acquisition, Supervision, Writing – original draft, Writing – review & editing. YC: Formal analysis, Investigation, Validation, Writing – original draft. HT: Methodology, Writing – review & editing. SW: Methodology, Writing – review & editing. XL: Methodology, Writing – review & editing. PH: Methodology, Writing – review & editing. JZ: Methodology, Writing – review & editing. KW: Methodology, Supervision, Writing – review & editing. YY: Formal analysis, Supervision, Writing – original draft, Writing – review & editing.

## References

[B1] Bao A. L. Burritt D. J. Chen H. F. Zhou X. N. Cao D. Tran L. P. (2019). The CRISPR/Cas9 system and its applications in crop genome editing. Crit. Rev. Biotechnol. 39, 321–336. doi: 10.1080/07388551.2018.1554621 30646772

[B2] Blakeslee A. F. Belling J. Farnham M. E. Bergner A. D. (1922). A haploid mutant in the jimson weed, “Datura stramonium”. Science 55, 646–647. doi: 10.1126/science.55.1433.646 17734425

[B3] Castillo A. M. Valero-Rubira I. Allue S. Costar M. A. Valles M. P. (2021). Bread wheat doubled haploid production by anther culture. Methods Mol. Biol. 2287, 227–244. doi: 10.1007/978-1-0716-1315-3_11 34270033

[B4] Chaikam V. Nair S. K. Martinez L. Lopez L. A. Utz H. F. Melchinger A. E.. (2018). Marker-assisted breeding of improved maternal haploid inducers in maize for the tropical/subtropical regions. Front. Plant Sci. 9, 1527. doi: 10.3389/fpls.2018.01527 30405665 PMC6201356

[B5] Chen C. Liu X. Q. Li S. Z. Liu C. X. Zhang Y. L. Luo L. L.. (2022). Co-expression of transcription factors *ZmC1* and *ZmR2* establishes an efficient and accurate haploid embryo identification system in maize. Plant J. 111, 1296–1307. doi: 10.1111/tpj.15888 35793378

[B6] Cheng Z. X. Sun Y. Yang S. H. Zhi H. Yin T. Ma X. J.. (2021). Establishing in planta haploid inducer line by edited *SiMTL* in foxtail millet (*Setaria italica*). Plant Biotechnol. J. 19, 1089–1091. doi: 10.1111/pbi.13584 33749085 PMC8196629

[B7] Cistué L. Romagosa I. Batlle F. Echávarri B. (2009). Improvements in the production of doubled haploids in durum wheat (*Triticum turgidum* L.) through isolated microspore culture. Plant Cell Rep. 28, 727–735. doi: 10.1007/s00299-009-0690-6 19288107

[B8] Dong L. Li L. N. Liu C. L. Liu C. X. Geng S. F. Li X. H.. (2018). Genome editing and double-fluorescence proteins enable robust maternal haploid induction and identification in maize. Mol. Plant 11, 1214–1217. doi: 10.1016/j.molp.2018.06.011 30010025

[B9] Eliby S. Bekkuzhina S. Kishchenko O. Iskakova G. Kylyshbayeva G. Jatayev S.. (2022). Developments and prospects for doubled haploid wheat. Biotechnol. Adv. 60, 108007. doi: 10.1016/j.biotechadv.2022.108007 35732257

[B10] Forster B. P. Heberle-Bors E. Kasha K. J. Touraev A. (2007). The resurgence of haploids in higher plants. Trends Plant Sci. 12, 368–375. doi: 10.1016/j.tplants.2007.06.007 17629539

[B11] Gan W. C. Ling A. P. K. (2022). CRISPR/Cas9 in plant biotechnology: applications and challenges. BioTechnologia (Pozn) 103, 81–93. doi: 10.5114/bta.2022.113919 36605382 PMC9642946

[B12] Han F. P. Liu B. Fedak G. Liu Z. H. (2004). Genomic constitution and variation in five partial amphiploids of wheat-*Thinopyrum intermedium* as revealed by GISH, multicolor GISH and seed storage protein analysis. Theor. Appl. Genet. 109, 1070–1076. doi: 10.1007/s00122-004-1720-y 15197444

[B13] Ishii T. Karimi-Ashtiyani R. Houben A. (2016). Haploidization via chromosome elimination: means and mechanisms. Annu. Rev. Plant Biol. 67, 421–438. doi: 10.1146/annurev-arplant-043014-114714 26772657

[B14] Jacquier N. M. A. Gilles L. M. Pyott D. E. Martinant J. P. Rogowsky P. M. Widiez T. (2020). Puzzling out plant reproduction by haploid induction for innovations in plant breeding. Nat. Plants 6, 610–619. doi: 10.1038/s41477-020-0664-9 32514145

[B15] Karimi-Ashtiyani R. (2021). Centromere engineering as an emerging tool for haploid plant production: advances and challenges (New York: Springer US). 10.1007/978-1-0716-1331-3_1 34270060

[B16] Kelliher T. Starr D. Richbourg L. Chintamanani S. Delzer B. Nuccio M. L.. (2017). MATRILINEAL, a sperm-specific phospholipase, triggers maize haploid induction. Nature 542, 105–109. doi: 10.1038/nature20827 28114299

[B17] Kelliher T. Starr D. Su X. J. Tang G. Z. Chen Z. Y. Carter J.. (2019). One-step genome editing of elite crop germplasm during haploid induction. Nat. Biotechnol. 37, 287–292. doi: 10.1038/s41587-019-0038-x 30833776

[B18] Li Y. Lin Z. Yue Y. Zhao H. M. Fei X. H. Lizhu E.. (2021). Loss-of-function alleles of *ZmPLD3* cause haploid induction in maize. Nat. Plants 7, 1579–1588. doi: 10.1038/s41477-021-01037-2 34887519 PMC8677622

[B19] Li J. F. Norville J. E. Aach J. McCormack M. Zhang D. Bush J.. (2013). Multiplex and homologous recombination–mediated genome editing in *Arabidopsis* and *Nicotiana benthamiana* using guide RNA and Cas9. Nat. Biotechnol. 31, 688–691. doi: 10.1038/nbt.2654 23929339 PMC4078740

[B20] Liu H. Y. Ke W. Jia Z. M. Gong Q. Lin Z. S. Du L. P.. (2020b). Efficient induction of haploid plants in wheat by editing of *TaMTL* using an optimized *Agrobacterium*-mediated CRISPR system. J. Exp. Bot. 71, 1337–1349. doi: 10.1093/jxb/erz529 31760434 PMC7031065

[B21] Liu C. X. Li X. Meng D. X. Zhong Y. Chen C. Dong X.. (2017). A 4-bp insertion at *ZmPLA1* encoding a putative phospholipase a generates haploid induction in maize. Mol. Plant 10, 520–522. doi: 10.1016/j.molp.2017.01.011 28179149

[B22] Liu C. X. Zhong Y. Qi X. L. Chen M. Liu Z. K. Chen C.. (2020a). Extension of the in *vivo* haploid induction system from diploid maize to hexaploid wheat. Plant Biotechnol. J. 18, 316–318. doi: 10.1111/pbi.13218 31344311 PMC6953200

[B23] Long L. Feng Y. M. Shang S. Z. Zhao J. R. Hu G. Y. Xu F. C.. (2024). *In vivo* maternal haploid induction system in cotton. Plant Physiol. 194, 1286–1289. doi: 10.1093/plphys/kiad620 37979158

[B24] Lv J. Yu K. Wei J. Gui H. P. Liu C. X. Liang D. W.. (2020). Generation of paternal haploids in wheat by genome editing of the centromeric histone CENH3. Nat. Biotechnol. 38, 1397–1401. doi: 10.1038/s41587-020-0728-4 33169035

[B25] Meng D. X. Liu C. X. Chen S. J. Jin W. W. (2021). Haploid induction and its application in maize breeding. Mol. Breed. 41, 20. doi: 10.1007/s11032-021-01204-5 37309420 PMC10236068

[B26] Mulugeta B. Ortiz R. Geleta M. Hailesilassie T. Hammenhag C. Hailu F.. (2023). Harnessing genome-wide genetic diversity, population structure and linkage disequilibrium in Ethiopian durum wheat gene pool. Front. Plant Sci. 14, 1192356. doi: 10.3389/fpls.2023.1192356 37546270 PMC10400094

[B27] Nair S. K. Molenaar W. Melchinger A. E. Boddupalli P. M. Martinez L. Lopez L. A.. (2017). Dissection of a major QTL *qhir1* conferring maternal haploid induction ability in maize. Theor. Appl. Genet. 130, 1113–1122. doi: 10.1007/s00122-017-2873-9 28315926 PMC5440511

[B28] Puchta H. Dujon B. Hohn B. (1996). Two different but related mechanisms are used in plants for the repair of genomic double-strand breaks by homologous recombination. Proc. Natl. Acad. Sci. U. S. A. 93, 5055–5060. doi: 10.1073/pnas.93.10.5055 8643528 PMC39405

[B29] Qi X. L. Guo S. W. Zhong Y. Chen B. J. Liu Z. K. Yan T. Z.. (2023). Establishment of an efficient haploid identification system by engineering anthocyanin accumulation in the wheat embryo. Plant Commun. 4, 100568. doi: 10.1016/j.xplc.2023.100568 36864726 PMC10203450

[B30] Sari N. Solmaz I. (2021). Doubled haploid production in watermelon. Methods Mol. Biol. 2289, 97–110. doi: 10.1007/978-1-0716-1331-3_6 34270065

[B31] Shimatani Z. Kashojiya S. Takayama M. Terada R. Arazoe T. Ishii H.. (2017). Targeted base editing in rice and tomato using a CRISPR-Cas9 cytidine deaminase fusion. Nat. Biotechnol. 35, 441–443. doi: 10.1038/nbt.3833 28346401

[B32] Tang H. L. Wang K. Zhang S. X. Han Z. Y. Chang Y. N. Qiu Y. L.. (2023). A fast technique for visual screening of wheat haploids generated from *TaMTL*-edited mutants carrying anthocyanin markers. Plant Commun. 4, 100569. doi: 10.1016/j.xplc.2023.100569 36864725 PMC10203451

[B33] Tang H. L. Zhang S. X. Yu M. Wang K. Yu Y. Qiu Y. L.. (2022). Effects of *TaMTL*-edited mutations on grain phenotype and storage component composition in wheat. Agriculture 12, 587. doi: 10.3390/agriculture12050587

[B34] Tian X. L. Qin Y. X. Chen B. J. Liu C. X. Wang L. L. Li X. L.. (2018). Hetero-fertilization together with failed egg-sperm cell fusion supports single fertilization involved in *in vivo* haploid induction in maize. J. Exp. Bot. 69, 4689–4701. doi: 10.1093/jxb/ery177 29757396 PMC6137981

[B35] Tian S. W. Zhang J. Zhao H. Zong M. Li M. Y. Gong G. Y.. (2023). Production of double haploid watermelon via maternal haploid induction. Plant Biotechnol. J. 21, 1308–1310. doi: 10.1111/pbi.14045 36951091 PMC10281598

[B36] Wang K. Liu H. Y. Du L. P. Ye X. G. (2017). Generation of marker-free transgenic hexaploid wheat via an *Agrobacterium*-mediated co-transformation strategy in commercial Chinese wheat varieties. Plant Biotechnol. J. 15, 614–623. doi: 10.1111/pbi.12660 27862820 PMC5399001

[B37] Weyen J. (2021). Applications of doubled haploids in plant breeding and applied research. Methods Mol. Biol. 2287, 23–39. doi: 10.1007/978-1-0716-1315-3_2 34270024

[B38] Wu J. Yin H. (2019). Engineering guide RNA to reduce the off-target effects of CRISPR. J. Genet. Genomics 46, 523–529. doi: 10.1016/j.jgg.2019.11.003 31902584

[B39] Yao L. Zhang Y. Liu C. X. Liu Y. B. Wang Y. L. Liang D. W.. (2018). *OsMATL* mutation induces haploid seed formation in indica rice. Nat. Plants 4, 530–533. doi: 10.1038/s41477-018-0193-y 29988153

[B40] Yin M. Q. Zhang S. X. Fan C. K. Wang K. Y. Wang J. Wang K.. (2018). Effects of different chemicals and treatment methods on chromosome doubling of haploid wheat plants. Sci. Agric. Sin. 51, 811–820. doi: 10.3864/j.issn.0578-1752.2018.05.001

[B41] Yuan J. Guo X. Hu J. Lv Z. L. Han F. P. (2015). Characterization of two *CENH3* genes and their roles in wheat evolution. New Phytol. 206, 839–851. doi: 10.1111/nph.13235 25557089

[B42] Zhang J. Z. Yin J. Luo J. Y. Tang D. Zhu X. J. Wang J.. (2022). Construction of homozygous diploid potato through maternal haploid induction. aBIOTECH 3, 163–168. doi: 10.1007/s42994-022-00080-7 36304841 PMC9590536

[B43] Zhong Y. Chen B. J. Li M. R. Wang D. Jiao Y. Y. Qi X. L.. (2020). A DMP-triggered in *vivo* maternal haploid induction system in the dicotyledonous *Arabidopsis* . Nat. Plants 6, 466–472. doi: 10.1038/s41477-020-0658-7 32415294

[B44] Zhong Y. Chen B. J. Wang D. Zhu X. J. Li M. R. Zhang J. Z.. (2022). *In vivo* maternal haploid induction in tomato. Plant Biotechnol. J. 20, 250–252. doi: 10.1111/pbi.13755 34850529 PMC8753351

[B45] Zhong Y. Liu C. X. Qi X. L. Jiao Y. Y. Wang D. Wang Y. W.. (2019). Mutation of *ZmDMP* enhance haploid induction in maize. Nat. Plants 5, 575–580. doi: 10.1038/s41477-019-0443-7 31182848

[B46] Zong Y. Wang Y. P. Li C. Zhang R. Chen K. L. Ran Y. D.. (2017). Precise base editing in rice, wheat and maize with a Cas9-cytidine deaminase fusion. Nat. Biotechnol. 35, 438–440. doi: 10.1038/nbt.3811 28244994

